# The Challenge of Greening Religious Schools by Improving the Environmental Competencies of Teachers

**DOI:** 10.3389/fpsyg.2020.00520

**Published:** 2020-03-24

**Authors:** Rafael Robina-Ramírez, M. Isabel Sánchez-Hernández, Héctor V. Jiménez-Naranjo, Carlos Díaz-Caro

**Affiliations:** ^1^Department of Business Organization and Sociology, School of Business and Tourism, Cáceres, Spain; ^2^Department of Business Organization and Sociology, School of Economics and Business Administration, University of Extremadura, Badajoz, Spain; ^3^Department of Finance and Accounting, School of Business, Finance and Tourism, Cáceres, Spain

**Keywords:** competences, environmental threat, greening, schools, religious schools

## Abstract

Even though sacred scriptures emphasize the key role that Creation and respect for living creatures play in all religions, the so-called religious schools seem to show little interest in putting this sacred mandate into effect. To shed light on this subject, this work investigates the role of teachers in the process, focusing on their environmental competencies. Our hypotheses are tested through a structural equation model on a sample of 214 biology and religion teachers from 118 Catholic schools in Spain who voluntary participated in a survey. The research findings confirm that it is crucial that environmental competencies are developed in teachers to enable the greening of schools. Theoretical and practical implications for defining the job training of teachers in religious schools are drawn from the study.

## Introduction

Recently, the World Economic Forum’s *Global Risks Report 2018* has warned about some of the biggest environmental threats in the near future, namely extreme weather events and natural disasters, water crises, biodiversity loss, and air and soil pollution (Hossain and Purohit, 2018). These challenging threats have previously been defined as ‘wicked problems’ (Rittel and Webber, 1973) because of the difficulties in finding optimal solutions to them (Shindler and Cramer, 1999).

In the last three decades, a vast amount of literature has been published with the aim of tackling these environmental threats (Van Eijndhoven et al., 2001; Farrell and Jäger, 2006). Despite the outstanding efforts made by environmental organizations to preserve nature, it is broadly agreed that natural resources are still not used and replaced appropriately (Melkert and Vos, 2008; World Health Organization [WHO], 2013) because social, economic and environmental resources are not developed in harmony (Kates et al., 2005). Economic development and environmental needs and resources should therefore be reconciled (Kuhlman and Farrington, 2010).

Sustainable development has been defined and identified as the preservation of natural resources (the environmental perspective) but also as cooperation between communities (the socio-economic perspective) (Rauch, 2002). As the Brundtland Commission described, sustainable development can be developed by meeting the needs of the current times and by respecting future generations (WCED, 1987). The sustainable methodology that links economic development and the environment needs to be taught at an early stage in life. As the UNECE (2005) has stated, ‘it is important to ensure that all pupils and students acquire appropriate knowledge of sustainable development and are aware of the impact of decisions that do not support sustainable development’ (p. 6).

In this process of education in sustainability, schools have become the appropriate educational institutions to train new generations to use natural resources appropriately (Vare and Scott, 2007). In 1990, the government of Spain passed a national law known as the LOGSE (*General Organic Law about the Education System in Spain*), which introduced reforms in environmental education into the school curriculum. There is some evidence that from this time on schools in Spain have, slowly but gradually, increased their sustainability strategies for protecting the environment (Murga-Menoyo, 2009).

Similarly, regional governments have made environmental commitments to future generations by developing initiatives in sustainability such as the *Basque Strategy for Sustainable Environmental Development* (Government of the Basque Country, 2002) and the *Plan of Education for Sustainability* (Government of Cantabria, 2005). These plans include specific environmental initiatives that have already been applied in other educational institutions, such as, among others, using public transport instead of cars, or turning off lights when they are not being used, to decrease overall consumption (Shwom and Lorenzen, 2012).

Education in sustainability is also connected with the sacred scriptures (Northcott, 2009; Delio, 2017). It is based on the experience of the natural beauty of Creation, which triggers spiritual feelings of fascination and admiration (Palmer, 2008) that are directly connected to the protection of nature (Johnson, 2002). In this regard, Christianity has inspired the principle of the stewardship of nature (Boff, 1995); from the very beginning, ‘God saw all that he had made, and it was very good’ (Gen, 1:31); in the Greek version, the word ‘good’ is ‘kalon,’ meaning beautiful. Living creatures as well as human beings were made as beautiful things in the ‘image of God’ or the very likeness of the Creator (Gen, 1:27).

According to Hitzhusen (2006), religious elements enhance education in sustainability because of religion’s environmental values. Likewise, religion has the potential to teach an understanding of the process of life and living creatures as an ontological gift, as an example of a respectful attitude toward nature (Farrior and Lowry, 2001). Nevertheless, to the best of our knowledge, few studies until now have highlighted the challenge of greening religious schools to face global environmental threats. This paper aims to address this challenge by studying the variables that have a positive influence on the challenge of greening religious schools to face the global environmental risk, and the role of teachers’ competencies in relation to this.

### Literature Review and Development of Hypotheses

#### The Lack of Education in Sustainability in Religious Schools

Religious education in schools has traditionally addressed moral issues in order to help students to develop their own views by reflecting on how moral issues have a positive influence on behavior (Schreiner, 2000). The process of understanding moral issues in religious schools not only provides students with feelings of affiliation and of belonging to a religion, but also produces a moral atmosphere (Francis, 1986; Flynn, 1995), which gives students a sense of direction beyond materialistic approaches to life (Ysseldyk et al., 2010).

Since the Tbilisi Declaration (UNESCO, 1977) and the UN Conference on Environment and Development (Agenda 21), knowledge, values, attitudes and practical skills have been introduced into some European countries to solve environmental problems through teaching in schools. Aligned with those international regulations, religious schools in Finland, for instance, have included ‘responsibility for the environment, well-being and a sustainable future’ in their current national curriculum (Aarnio-Linnanvuori, 2013). Likewise, Indonesian religious schools, whose aim is to produce religious individuals and responsible citizens by being self-sufficient in natural resources, have followed the same path (Parker, 2017).

In Spain, the aforementioned LOGSE (1990) introduced compulsory environmental education in 1990. As a result, through Agenda 21, schools were monitored in their policies for developing initiatives toward sustainability (UN, 2009). Regions such as the Basque country, Cantabria, the Community of Madrid, Catalonia and the Balearic Islands were pioneers in defining the environmental content to be taught in schools (Murga-Menoyo, 2009).

Among these environmental initiatives, a network of affiliated eco-schools was set up in Spain. This was called the Association for Environmental Education and Consumers (ADEAC, 2019). Nowadays, 519 schools in Spain are integrated into this non-profit organization, which implements programs from the European Foundation for Environmental Education (FEE) (Parris, 2002). As the director of this altruistic organization has recently reported to the research team working on this paper, only approximately 4% of the schools in the network are religious schools. Thinking about the future generations of students in religious schools in Spain, the small size of that impact has driven us to find out which attributes might cause the greening of religious schools.

#### The Two Approaches to Teaching Sustainability in Schools

Religious and environmental connections (REC) exist. In the last decade, environmentalists and religious leaders have created overlapping and mixed relationships to raise environmental awareness, with the aim of protecting and preserving natural resources (Sponsel, 2012; Raven, 2016). However, the process of integrating religious rules into respectful attitudes has been complex.

Current environmental damage provoked by human beings has caused a destructive model of growth in developed and developing countries, and this has been denounced by religious leaders (Pope Paul, 1971; Benedict, 2008). More recently, Pope Francis, in the encyclical letter *Laudato Si*, has stressed the devastating consequences of this damage not only for the environment but also for human beings, namely in worldwide poverty (Raven, 2016).

Despite these environmental–religious statements, environmentalists and religious leaders have not yet found an amicable agreement that allows them to build one discourse upon the same values and attitudes (Biel and Nilsson, 2005), even though they share those values and attitudes (Crossman, 2011). On the religious side, rules to respect nature were set within the covenant between the Creator and the creatures (Berry, 1988), in order to shape attitudes and actions to protect and restore the environment (Tucker, 2009). From the side of environmentalists, environmental education is based on the same respectful values toward nature (Hitzhusen, 2005). To heal the disagreement with religious schools is key, not only to connecting environmental concepts and understanding why the protection of nature is part of the spiritual covenant (Dudley et al., 2009), but also for avoiding clashes and connecting sustainability and religion by efficiently using their common language (Gookin, 2002).

Hence, the justification of the importance of environmental education as a model of education in values is based on the impossibility in religious schools of maintaining a disagreement between religion, humanity and nature (Jensen and Schnack, 2006). After expressing the relationship between religious doctrine and sustainability in religious schools, we formulate the following hypothesis:

Hypothesis 1 (H1): The connection between religious doctrine and sustainability in religious schools (REC) positively influences the greening process of religious schools (GRS).

#### Sustainability as a Cross-Sectional Competence

Education in sustainability has currently become a challenge for schools. Schools, in particular, understand the benefits that lie between human development and the preservation of nature, and between the moral dimension of human beings and the environmental role that men and women play on earth. The purpose of education in sustainability is to develop environmental cross-sectional competences (CECs).

According to Jensen and Schnack (2006), highly educated individuals usually show moral behavior to promote their personal integrity. To preserve integrity among students, CSCs are aimed at developing knowledge, skills and rules of behavior (Loe Spanish National Education System, 2006). These competences are linked not only to social and ethical commitment (Haynes, 2002; Yildirim and Baştuğ, 2010), but also to proactive behavior (Clunies-Ross et al., 2008) that confronts the exploitation of natural resources (Mogensen and Mayer, 2005). However, according to Nekhoroshkov (2016), providing adequate knowledge for students is not sufficient to give them environmental competences.

Environmental knowledge, skill and a willingness to act responsibly toward nature (Torkar and Krašovec, 2019) have to be surrounded by an environmental awareness at school (Goldman et al., 2018; Olsson et al., 2019; Sánchez-Llorens et al., 2019) if the environment is to be protected. These elements help students to be passionate about nature (Uzzell et al., 1995). An ecological culture, a system that prioritizes the relationship between humans and nature, needs to be spread across society (Boulet et al., 2015). Hence, competences in environmental education do not only give protection to the student’s own social and natural environmental safety (Hashim and Denan, 2015; Ponomarenko et al., 2016).

To prioritize the connection between human beings and nature, education in sustainability has to develop systemic and holistic thinking (Lozano, 2006) to connect the environment with social and economic development (Rauch, 2002) and, as well, critical arguments to defend nature from voracious consumerism (Singseewo, 2011).

Taking into account what is expressed in this section, we formulate the following hypotheses:

Hypothesis 2 (H2): Cross-sectional environmental competence (CEC) positively influences the connection between religious doctrine and sustainability in religious schools (REC).

Hypothesis 3 (H3): Cross-sectional environmental competence (CEC) positively influences the greening process of religious schools (GRS).

### Environmental Teaching Programs (ETP)

Teaching environmental education in schools has played a key role in the educational process of turning young people into responsible citizens (Pascual et al., 2000). Environmental teaching programs (ETP) based on environmental policies help to transform local societies and communities (Pitoska and Lazarides, 2013; Rickenbacker et al., 2019).

This training should include ETP (Fien et al., 2008) as well as empirical ones (Gill and Lang, 2018; Robina-Ramírez and Medina-Merodio, 2019). It allows environmental policies to be developed that incorporate the appropriate interdisciplinary skills, competences and values to transform current society into a better environment (Heyl et al., 2013; Hofman, 2015).

According to Fien et al. (2008), this interdisciplinary method should express a social-environmental model rather than a basically educational model (Vázquez and Sevillano García, 2011). This means that ‘training for action’ and ‘social and environmental change’ need to be applied inside and outside schools (Heyl et al., 2013; Collins, 2017).

Empirical teaching and learning is based on experience, as educational policies focus not only on cognitive but also on affective processes in order to predispose students to assimilate this training (Ramos et al., 2015). ETP should also include affective emotions. There is no one-to-one correspondence between attitude and behavior unless moral emotions are included (Johnson and Manoli, 2011). Emotions, behaviors and values are deeply connected to religious and environmental education (Batson et al., 1985; Kals et al., 1999; Fletcher et al., 2005; Robina-Ramírez and Pulido Fernández, 2018).

Hence, teachers have to assess students’ attitudes based on the knowledge that the students have (Okur-Berberoglu, 2015), and, with the help of affective emotions, to engage with social and environmental goals for the students. As a result, knowledge and emotional and behavioral responses lead students to transform society through practical cases. Applying only traditional educational programs will only make students aware of the problem but will not show them how to act to solve environmental problems, whether these are global or local (Uzzell et al., 1995).

Environmental damage to nature can be local or international. According to Ideland and Malmberg (2015), the process of environmental education in religious schools must focus on the realities of the local communities and the environmental problems at the schools themselves in order to minimize the economic and environmental impact (Afrinaldi et al., 2017).

Taking into account what has been stated in this section, we formulate the following hypotheses:

Hypothesis 4 (H4): Cross-sectional environmental competence (CEC) positively influences environmental teaching programs in religious schools (ETP).

Hypothesis 5 (H5): Environmental teaching programs in religious schools (ETP) positively influence the greening process in religious schools (GRS).

#### Environmental Competencies of Teachers (ECTs)

The importance of human resource (HR) qualifications for educational institutions in general and for religious schools in particular has been a recurrent theme for several years now. More concretely, teacher training has become a key issue in introducing environmental education into schools, as we have been repeatedly reminded by international organizations (UNESCO, 1977). Training has been defined as a priority in order to promote sustainability (Fien, 1995).

Strategies to improve environmental education in society should be carried out with the effective support of teachers (Bregeon et al., 2008), or new generations will not transform their mind-sets and take up a respectful attitude to nature (Madhawa Nair et al., 2013). Teachers’ environmental knowledge plays a key role in developing the environmental competencies of their students (Mat Said et al., 2003; Guven and Sulun, 2017).

For instance, a study conducted in five schools in Canada showed that the level of teaching of environmental education in schools is not adequate (Miles et al., 2006). Other studies have highlighted that teachers do not have accurate knowledge about environmental literacy and competencies (Tal, 2010). These results were confirmed by the work of Falkenberg and Babiuk (2014), which stressed the low level of environmental knowledge and competences of teachers. This implies there is a need for a defined theoretical and practical learning process in environmental education to train these future educators. However, and in combination with this lack of environmental knowledge and competencies among teachers, complementary works have shown that teachers have positive attitudes toward environmental training (Fien and Tilbury, 1996; Zembylas, 2007).

Based on these positive attitudes, training courses have been applied to study their impact on students. Van Petegem et al. (2007) implemented environmental actions in two universities in the Netherlands with a high and a low level of environmental education. In the second one, the teachers trained the students poorly because of their lack of environmental education. Similar results were found by Valderrama-Hernández et al. (2017): teachers lacked sufficient knowledge and skills to teach their students, because of a lack of training.

Taking into account what has been said in this section, we formulate the following hypotheses:

Hypothesis 6 (H6): Environmental competencies of teachers (ECTs) positively influence cross-sectional environmental competence (CECs).

Hypothesis 7 (H7): Environmental competencies of teachers (ECTs) positively influence religious and environmental connections (RECs).

Hypothesis 8 (H8): Environmental competencies of teachers (ECTs) positively influence environmental teaching programs in religious schools (ETPs).

### Empirical Study

#### Model and Measures

**FIGURE 1:**
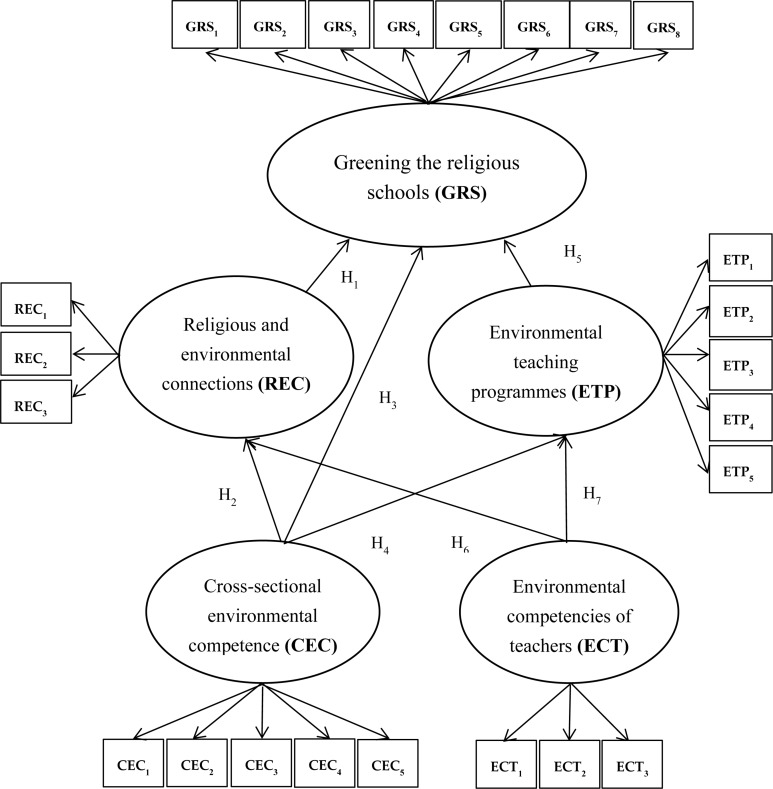
Conceptual model.

From the review of the literature, four constructs (REC, CEC, ETP, and ECT) was proposed to measure their impact on greening in religious schools (GRSs). The model is presented in Figure 1.

These constructs were designed with the objective of establishing the questionnaire items around the concepts proposed by different authors (see Table 1). The questions in the survey were measured using a Likert scale with seven points to indicate the degree of importance of the factors (Allen and Seaman, 2007). The factors or constructions were measured from 1 (‘strongly disagree’) to 7 (‘strongly agree’).

**TABLE 1:** Scales.

**Construct**	**Code**	**Item**	**Sources**
**Greening religious schools (GRSs)**	GRS1	Students’ general environmental knowledge	UNECE, 2005
	GRS2	Students’ environmental skills to tackle environmental threats	Goldman et al., 2018; Olsson et al., 2019
	GRS3	Students’ behavior to protect nature	Hashim and Denan, 2015; Robina-Ramírez and Fernández Portillo, 2018; Sánchez-Llorens et al., 2019
	GRS4	Students’ critical thinking about the environment	Singseewo, 2011
	GRS5	Students’ holistic and systematic thinking about the environment	Lozano, 2006; Svanström et al., 2008
	GRS6	Students’ specific environmental actions at school	Ward et al., 2014
	GRS7	Students’ proactive thinking about the environment	Clunies-Ross et al., 2008
	GRS8	Students’ social and ethical commitment to the environment	Haynes, 2002; Yildirim and Baştuğ, 2010
**Religious and environmental connections (RECs)**	REC1	Connect sustainability and teaching of sacred scriptures through examples	Hitzhusen, 2006
	REC2	Enhance common values in sustainability and religion	Meyfroidt, 2013; Robina-Ramírez and Pulido-Fernández, 2019
	REC3	Combine religious and sustainable activities at school	Tucker, 2009
**Cross-sectional environmental competence (CEC)**	CEC1	Train students to protect nature in class through critical thinking	Morrison et al., 2015
	CEC2	Deliver talks in class about environmental injustice and inequalities	Jensen and Schnack, 2006
	CEC3	Train students to acquire environmental values	Biel and Nilsson, 2005
	CEC4	Train students to be passionate about nature to avoid environmental threats	Uzzell et al., 1995
	CEC5	Bring local examples to class about the environment to raise students’ concerns	Palmer, 2008
**Environmental teaching programs (ETP)**	ETP1	Promote learning programs among students	Gill and Lang, 2018
	ETP2	Apply environmental education to local communities	Robina-Ramírez and Medina-Merodio, 2019; Robina-Ramírez et al., 2020
	ETP3	Developing teaching programs to assess the socio-economic impact on the environment	Afrinaldi et al., 2017
	ETP4	Address environmental education toward social change for students	Collins, 2017
	ETP5	Develop affective approach through environmental learning	Kals et al., 1999
**Environmental competencies of teachers (ECT)**	ECT1	Teachers have enough environmental knowledge	Mat Said et al., 2003; Guven and Sulun, 2017
	ECT2	Teachers have a positive attitude toward nature	Fien and Tilbury, 1996; Zembylas, 2007
	ECT3	Teachers develop skills to set up environmental strategies among students	Fien, 1995; Valderrama-Hernández et al., 2017

The questionnaire was first validated through qualitative interviews with teachers, six of them face-to-face and 15 in Skype calls. As a result of this validation process, three questions were modified to ensure that the teachers had the correct understanding.

#### Population and Sample

According to the Catholic Schools Organization, there are 1,996 Catholic schools in Spain. They educate 1,217,674 students and have a total of 83,352 teachers of region and biology.

In this study our hypotheses were tested with a convenience sample of teachers from 118 religious schools. Through telephone calls and emails, information was collected from 214 teachers from each of the 17 autonomous regions of Spain, between 1^st^ May and 10^th^ July 2019. 57% of the respondent were males, with the predominant age (70% of the total sample) being between 36 and 55. Most of them had been teaching for between 11 and 20 years, predominantly in secondary schools (see Table 2).

**TABLE 2:** Sample characterization.

**Attributes**	***N* = 214**	**Percentage (%)**
**Gender**		
Male	123	57
Female	91	43
**Total**	214	100
**Age**		
Less than 25	5	2
26–35	41	19
36–45	72	34
46–55	78	36
55 forward	18	8
**Total**	214	100
**Teaching experience**		
Less than 3	9	4
From 3 to 10	77	36
From 11 to 20	87	41
More than 20 years	41	19
**Total**	214	
**Subject**	214	
Biology	101	47
Religion	113	53
**Total**	214	100
Institution		
Primary education	78	36
Secondary education	92	43
Bachelor	44	21
**Total**	214	100
Socio-economic standard		
Low class	12	6
Middle class- High class	212	99
High class	0	0
**Total**	214	100

#### Method and Techniques

Partial least squares (PLS) structural equation modeling (SEM) is used for conceptual model design through causal and non-parametric predictive analysis (Hussain et al., 2018). It is, especially when based on a variance model, suitable for analyzing quantitative data in the areas of social sciences and organizational behavior (Fornell and Bookstein, 1982; Hair et al., 2012).

The data obtained from the *ad hoc* questionnaire were analyzed using SmartPLS 3, which is particularly recommended for composite models or constructions (Rigdon et al., 2017). This PLS statistical technique is applied when the data are structured in a series of interrelated dependency relationships between latent variables and indicators (Sarstedt et al., 2016). SmartPLS software 3.2.8 was used (Ringle et al., 2015).

## Results

### Results of the Measurement Model

The PLS approach is defined by two steps: the measurement model and the structural model evaluation. To elaborate the measurement model, we need to study the reliability and validity of the indicators in relation to the latent variables or constructs (Hair et al., 2016). We therefore analyzed the individual loads (λ) or simple correlations of the measures with their respective latent variables (λ ≥ 0.7 is accepted). Some indicators presented λ < 0.7, so they were deleted from the model (these λ values were the following: GRS5 = 0.516, GRS6 = 0.661, GRS7 = 0.585, CEC2 = 0.346, CEC5 = 0.557, ETP2 = 0.375, ETP5 = 0.667).

The Cronbach coefficient was used as an index of the reliability of the latent variables. The convergent validity of the latent variables was evaluated through the inspection of the average variance extracted (AVE) (accepted if > 0.5). Table 3 also shows that the square root of the average variance extracted (AVE) for each construct is greater than its highest correlation with any other construct.

**TABLE 3:** Validity and reliability.

**Latent variables**	**Indicator**	**Loadings**	**Cronbach’s alpha**	**Rho_A (Dijkstra-Henseler)**	**Composite reliability**	**Average variance extracted (AVE)**
CEC	CEC1	0.823	0.877	0.877	0.876	0.702
	CEC3	0.811				
	CEC4	0.877				
ECT	ECT1	0.922	0.923	0.924	0.923	0.800
	ECT2	0.891				
	ECT3	0.870				
ETP	ETP1	0.835	0.891	0.904	0.822	0.735
	ETP3	0.966				
	ETP4	0.758				
GRS	GRS1	0.814	0.923	0.924	0.923	0.705
	GRS2	0.892				
	GRS3	0.841				
	GRS4	0.829				
	GRS8	0.822				
REC	REC1	0.865	0.929	0.930	0.929	0.814
	REC2	0.926				
	REC3	0.915				

The discriminant validity of the latent variables was verified using the Fornell–Larcker criterion (Fornell and Bookstein, 1982), by examining whether the square root of the average extracted value (AVE) of each item was above the correlations with the other latent variables. In addition, following Henseler et al. (2015), a test was performed to check the lack of discriminant validity is better detected with another technique. This test is called the heterotrait–monotrait relationship (HTMT). Table 4 shows that the HTMT ratio for each pair of factors was less than 0.90 (Henseler, 2017).

**TABLE 4:** Discriminant validity.

	**Fornell–Larcker test**					**Heterotrait–monotrait ratio**				
						**(HTMT)**				
		
	**CEC**	**ECT**	**ETP**	**GRS**	**REC**	**CEC**	**ECT**	**ETP**	**GRS**	**REC**
CEC	**0.838**									
ECT	0.425	**0.895**				0.424				
ETP	0.770	0.505	**0.857**			0.769	0.502			
GRS	0.709	0.675	0.740	**0.850**		0.707	0.674	0.735		
REC	0.760	0.654	0.667	0.840	**0.902**	0.761	0.653	0.668	0.849	

*The discriminant validity was assessed by comparing the square root of each AVE in the diagonal with the correlation coefficients (off-diagonal) for each construct in the relevant rows and columns.*

### Results of the Structural Model

Once we had examined the measurement model, we analyzed the relationships between the latent variables. First, we studied the path coefficients relative to each of the hypotheses. For this we tested the model from 5,000 sub-samples in order to verify the statistical significance of each path. From this, we obtained the explained variance (*R*^2^) of the endogenous latent variables, and the *p*-values of the regression coefficients (*t*-test) were used as indicators of the explanatory power of the model (Table 5).

**TABLE 5:** Path coefficients and statistical significance.

**Hypotheses**		**β**	**2.5%**	**97.5%**	***t* Statistics**	***p*-values**
H_1_	REC→GRS	0.338	0.046	0.670	2.110	0.000***
H_2_	CEC→REC	0.588	0.430	0.735	7.600	0.000***
H_3_	CEC→GRS	−0.0.64	0.415	0.225	0.391	0.696
H_4_	CEC→ETP	0.678	0.541	0.808	9.698	0.000***
H_5_	ETP→GRS	0.342	0.115	0.688	2.342	0.019*
H_6_	ECT→CEC	0.425	0.192	0.624	3.824	0.000***
H_7_	ECT→REC	0.404	0.278	0.542	5.957	0.000***
H_8_	ECT→ETP	0.678	0.541	0.808	4.046	0.030*

*p < 0.05 [*t*(0.05; 499) = 1.647]; **p < 0.01 [*t*(0.01; 499) = 2.333]; ***p < 0.001 [*t*(0.001; 499) = 3.106] (*n* = 5000 subsamples).

Six of the eight hypotheses were accepted. Among the accepted hypotheses, there were no statistically significant differences in the relationships between the variables in our model (value of *p* > 0.05).

### Goodness-of-Fit Test for the Model

First, the overall fit of the model was evaluated using the mean residual standard square root (SRMR) indicator. According to Hu and Bentler (1998), the SRMR is the average mean squared discrepancy between the correlations observed and the implicit correlations in the model. For values lower than 0.08, the SRMR indicator is considered to be acceptable for PLS (Henseler et al., 2016). In this study, the SRMR was 0.057, which means that the model fits the empirical data (Hair et al., 2016).

According to Chin (1998), the *R*^2^ values obtained for the investigation have the following significance: 0.67 ‘Substantial,’ 0.33 ‘Moderate,’ and 0.19 ‘Weak.’ The result obtained for the principal dependent variable, the greening process of religious schools (GRS), was *R*^2^ = 77.8%. Therefore, the evidence shows that the model presented has a solid or substantial predictive capacity. The other endogenous variables are also relevant, with substantial and moderate predictive capacity (for REC, *R*^2^ = 0.712, and for ETP *R*^2^ = 0.632). However, CEC has a weak capacity for prediction (*R*^2^ = 0.181). This explains that REC and ETP are the factors that contribute to the greening process of religious schools (GRS).

The blindfolding technique consists of omitting some of the data for a given construct during the estimation of the parameters and then trying to estimate what was omitted from the estimated parameters (Chin, 1998). Using this technique, the predictive relevance of the model is studied, to demonstrate that the model has predictive capacity.

As can be seen in Table 6, all the endogenous constructs have *Q*^2^ > 0. In the Stone–Geisser (*Q*^2^) test (Geisser, 1974; Stone, 1974), the values are fixed in three steps; 0.02, 0.15 and 0.35, indicating small, medium and high predictive relevance. All our constructs have predictive relevance, since the values of *Q*^2^ are all greater than 0.02.

**TABLE 6:** Coefficient determination (*R*^2^) and Stone–Geisser test (*Q*^2^).

**Construct**	***R*^2^**	***Q*^2^**
CEC	0.181	0.109
ETP	0.632	0.374
GRS	0.778	0.478
REC	0.712	0.495

## Discusion

**FIGURE 2:**
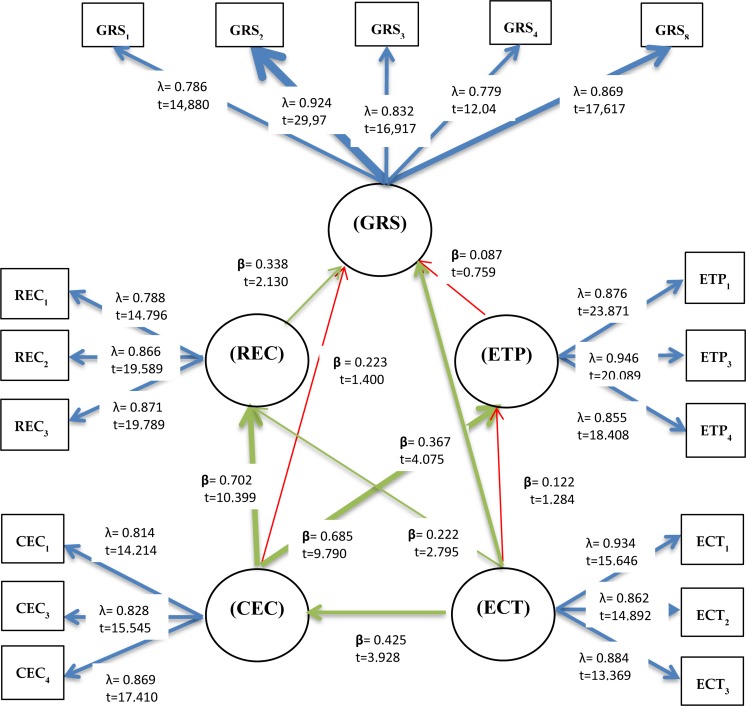
Results.

The paper discusses the environmental attributes that should be incorporated into religious schools to combine religious and environmental teaching. Figure 2 shows, in green, the hypotheses that were validated and, in red, the single hypothesis that was not accepted. The arrows are wider or narrower depending on the values of the supporting parameters (*t* student, *p*-values and path coefficients). The wider arrows link students’ environmental competencies (CEC) (through H2 and H4) and the environmental competencies of teachers (ECT) (through H4, H6 and H8) with the dependent variable. This demonstrates how knowledge and skill play a key role in greening religious schools.

From the results shown in Figure 2, we can say that there are two clear ways of greening religious schools. The first is by developing the cross-sectional environmental competences of students (CEC). The fact that H3 was not fulfilled means it is not possible to make religious schools green only on the basis of students’ competences and in the absence of overlapping religious and environmental teaching.

The second way, which is very important in the model as it is the only independent variable not directly affected by any other, is through improving teachers’ competences (ECT). Having training programs especially designed to improve the environmental skills of teachers in religious schools could be considered a good HR policy, and would have a direct positive impact on CEC, REC, and ETP and an indirect positive effect on GRS.

To sum up, 77.8% of the greening of religious schools is explained in the model through the selected constructs, where training programs for teachers are revealed as relevant (ECT). The environmental challenge for religious schools can be addressed by taking into account the connections between religion and the environment (REC) (*R*^2^ = 0.712) as well as environmental teaching programs (ETP) (*R*^2^ = 0.632) and cross-sectional environmental competences (*R*^2^ = 0.181). This model is strongly predictive, according to Chin and Newsted (1999).

The results obtained can allow decision-makers to design green strategies based on the role of these educational and religious variables in religious schools. In other words, it is worthwhile and highly recommended to introduce a common language based on the similar values between religion and environmental teaching among students. For this purpose, it would be necessary, first, to train teachers in environmental issues.

In addition, on the religious side, the model focuses on the relevance of Creation, encouraging students to consider their links with living creatures to enhance their commitment to environmental protection and preservation (Tucker, 1999). On the biology side, the model encourages the connection between the religious values of sacred scriptures and environmental science (Hungerford and Volk, 1990), in order to approach nature with respect in daily life (Kellert et al., 2002).

## Conclusion

As a result of the increase in environmental threats (Hossain and Purohit, 2018), scholars have focused on making environmental proposals to increase environmental awareness among the population (Van Eijndhoven et al., 2001; Farrell and Jäger, 2006). In this regard, sustainable development is playing a major role (Rauch, 2002).

As UNECE (2005) has recommended, sustainable development has to start to be taught in schools. Schools in Spain have started to be more aware of the role nature plays in education. However, religious schools are barely interested in this environmental teaching (ADEAC, 2019), which is incomprehensible if one takes into account the fact that Creation and living creatures are deeply rooted in sacred scriptures as well as in recent encyclical letters and religious documents (Pope Paul, 1971; Benedict, 2008). Several conclusions can be drawn from the results of this paper.

First, religious schools should combine religious teaching with environmental teaching (Sponsel, 2012; Raven, 2016). This teaching has to be based on the common values and knowledge taken from the sacred scriptures, in which Creation stories compel individuals to respect nature (Biel and Nilsson, 2005). Positive attitudes need to be built among teachers of biology and religion to enable them to speak the same language to students (Gookin, 2002). Consequently, the environmental training of teachers is a key issue in introducing environmental education to schools. HR managers in religious schools have to consider innovative programs to create or to reinforce the environmental competences of teachers. An environmental training policy, oriented toward human capital development, will lead to improved educational results and could also be considered as a differentiation strategy.

Second, teaching and learning programs have become an interesting tool to help raise environmental awareness in students. They have played a key role among teachers in biology and religion at schools. To make religious schools greener, it is crucial to develop not only CSCs (Mogensen and Mayer, 2005) but also teachers. These programs often give common guidance for students about right and wrong. Such competences need to be updated to cover environmental issues; the programs are usually based on critical thinking but also focus on students’ personal commitment (Lambrechts et al., 2013) and preparing them for action (Cincera and Krajhanzl, 2013). Similarly, they are usually based on rational attributes, without connections to affective reasons that would make students passionate about respecting nature (Uzzell et al., 1995).

Third, there is no direct way to make schools greener just by developing the students’ environmental competences; this must be done through combining religious and environmental teaching. In other words, students’ environmental competence needs to be mediated by combined religious and environmental teaching (Jensen and Schnack, 2006).

These results are aligned with the experiences of international programs for eco-schools that monitor schools’ designated plans (FEE, 2012). In the case of religious schools in Spain, it would be interesting to learn from international experience, because few of these religious schools are currently interested in greening their academic curriculum. Finally, the output of this research, even acknowledging the limitations derived from the peculiarities of the Spanish context, might shed light on other studies that find no relationship between religion and environmental science or precisely the opposite result (Kanagy and Nelsen, 1995; Clements et al., 2014; Morrison et al., 2015; Arbuckle, 2017). Because of the novelty of our research, this study could be considered as a starting point for future developments in new research contexts with complementary methods.

## Data Availability Statement

All datasets generated for this study are included in the article/supplementary material.

## Author Contributions

RR-R collected the data, define the methodology and wrote the manuscript. MS-H revised and corrected the manuscript. CD-C and HJ-N have both complemented the manuscript during the revision process.

## Conflict of Interest

The authors declare that the research was conducted in the absence of any commercial or financial relationships that could be construed as a potential conflict of interest.

## References

[B1] Aarnio-Linnanvuori E. (2013). Environmental issues in Finnish school textbooks on religious education and ethics. *Nordidactica* 1 131–157.

[B2] ADEAC (2019). *Association for the Environmental Education and Consumers.* Available online at: http://www.adeac.es [accessed July 15, 2019].

[B3] Afrinaldi F. Tasman A. M. Zhang H. C. Hasan A. (2017). Minimizing economic and environmental impacts through an optimal preventive replacement schedule: Model and application. *J. Clean. Prod.* 143 882–893. 10.1016/j.jclepro.2016.12.033

[B4] Allen I. E. Seaman C. A. (2007). Likert scales and data analyses. *Qual. Prog.* 40 64–65.

[B5] Arbuckle M. B. (2017). The interaction of religion, political ideology, and concern about climate change in the United States. *Soc. Nat. Resour.* 30 177–194. 10.1080/08941920.2016.1209267

[B6] Batson C. D. Schoenrade P. A. Pych V. (1985). Brotherly love or self-concern? Behavioural consequences of religion. *Adv. Psychol. Relig.* 11 185–208. 10.1016/b978-0-08-027948-0.50019-0

[B7] Benedict X. V. I. (2008). *Address to the Diplomatic Corps Accredited to the Holy See.* Rome: Libreria Editrice Vaticana.

[B8] Berry T. (1988). *The Dream of the Earth.* San Francisco, CA: Sierra Book Club.

[B9] Biel A. Nilsson A. (2005). Religious values and environmental concern: harmony and detachment. *Soc. Sci. Q.* 86 178–191. 10.1111/j.0038-4941.2005.00297.x

[B10] Boff L. (1995). *Liberation and Ecology.* Maryknoll, NY: Orbis Books.

[B11] Boulet M. Reid A. Emery S. Hill A. (2015). “New perspectives on research in environmental and sustainability education,” in *Proceedings of the 8th World Environmental Education Congress Planet and People*, Barcelona.

[B12] Bregeon J. Faucheux S. Rochet C. (2008). *Report of the Interdepartmental Working Group on Education for Sustainable Development].* Available online at: http://cache.media.education.gouv.fr (accessed July 11, 2019).

[B13] Chin W. W. (1998). “The partial least squares approach to structural equation modeling,” in *Modern Methods for Business Research*, ed. Marcoulides G. A. 295 (New York, NY: Lawrence Erlbaum Associates Publishers), 295–336.

[B14] Chin W. W. Newsted P. R. (1999). “Structural equation modelling analysis with small samples using partial least squares,” in *Statistical Strategies for Small Sample Research*, ed. Hoyle R. H. (Thousand Oaks, CA: Sage), 307–341.

[B15] Cincera J. Krajhanzl J. (2013). Eco-Schools: what factors influence pupils’ action competence for pro-environmental behaviour? *J. Clean. Prod.* 61 117–121. 10.1016/j.jclepro.2013.06.030

[B16] Clements J. M. Xiao C. McCright A. M. (2014). An examination of the “greening of Christianity” thesis among Americans, 1993–2010. *J. Sci. Study Relig.* 53 373–391. 10.1111/jssr.12116

[B17] Clunies-Ross P. Little E. Kienhuis M. (2008). Self-reported and actual use of proactive and reactive classroom management strategies and their relationship with teacher stress and student behaviour. *Educ. Psychol.* 28 693–710. 10.1080/01443410802206700

[B18] Collins T. J. (2017). Review of the twenty-three year evolution of the first university course in green chemistry: teaching future leaders how to create sustainable societies. *J. Clean. Prod.* 140 93–110. 10.1016/j.jclepro.2015.06.136

[B19] Crossman J. (2011). Environmental and spiritual leadership: tracing the synergies from an organizational perspective. *J. Bus. Ethics* 103 553–565. 10.1007/s10551-011-0880-3

[B20] Delio I. (2017). Is Natural Law “Unnatural?”. Exploring God and Nature Through Teilhard’s Organic Theology. *Theol. Sci.* 15 276–288. 10.1080/14746700.2017.1335063

[B21] Dudley N. Higgins-Zogib L. Mansourian S. (2009). The links between protected areas, faiths, and sacred natural sites. *Conserv. Biol.* 23 568–577. 10.1111/j.1523-1739.2009.01201.x 22748093

[B22] Falkenberg T. Babiuk G. (2014). The status of education for sustainability in initial teacher education programmes: a Canadian case study. *Int. J. Sustain. High. Educ.* 15 418–430. 10.1108/IJSHE-10-2012-0088

[B23] Farrell A. E. Jäger J. (eds) (2006). *Assessments of Regional and Global Environmental Risks. Designing Processes for the Effective Use of Science in Decision Making.* Washington. DC: RFF Press Book.

[B24] Farrior M. Lowry S. (2001). *Building Partnerships with the Faith Community: A Resource Guide for Environmental Groups.* Madison, WI: The Biodiversity Project.

[B25] FEE (2012). *Eco-schools.* Available online at: http://www.eco-schools.org/page.php?id1/452 (accessed February 10, 2019).

[B26] Fien J. (1995). Teaching for a sustainable world: the environmental and development education project for teacher education. *Environ. Educ. Res.* 1 21–33. 10.1080/1350462950010102

[B27] Fien J. Neil C. Bentley M. (2008). Youth can lead the way to sustainable consumption. *J. Educ. Sustain. Dev.* 2 51–60. 10.1177/097340820800200111

[B28] Fien J. Tilbury D. (1996). *Learning for a Sustainable Environment: An Agenda for Teacher Education in Asia and the Pacific.* Bangkok: UNESCO.

[B29] Fletcher T. Haynes J. Miller J. (2005). “Effects of grouping by perceived ability on the attitudes of year 10 students towards physical education,” in *Proceedings of the International Conference Australian Association for Research in Education (AARE)*, Sydney.

[B30] Flynn M. (1995). *The Culture of Catholic Schools: A Study of Catholic schools, 1972-1993.* Homebush, NSW: St Paul Publications.

[B31] Fornell C. Bookstein F. L. (1982). Two structural equation models: LISREL and PLS applied to consumer exit-voice theory. *J. Mark. Res.* 19 440–452. 10.1177/002224378201900406

[B32] Francis L. (1986). Roman Catholic secondary schools: falling rolls and pupil attitudes. *Educ. Stud.* 12 121–134.

[B33] Geisser S. (1974). A predictive approach to the random effect model. *Biometrika* 61 101–107. 10.1093/biomet/61.1.101

[B34] Gill C. Lang C. (2018). Learn to conserve: the effects of in-school energy education on at-home electricity consumption. *Energy Policy* 118 88–96. 10.1016/j.enpol.2018.03.058

[B35] Goldman D. Ayalon O. Baum D. Weiss B. (2018). Influence of ‘green school certification’on students’ environmental literacy and adoption of sustainable practice by schools. *J. Clean. Prod.* 183 1300–1313. 10.1016/j.jclepro.2018.02.176

[B36] Gookin J. (2002). “Spirituality: the softer side of education,” in *NOLS Environmental Education Notebook*, eds Gookin J. Wells D. (Lander, WY: NOLS), 7–8.

[B37] Government of Cantabria (2005). *Plan of Education for Sustainability.* Available online at: http://boc.gobcantabria.es [accessed February 21, 2019].

[B38] Government of the Basque Country (2002). *The Basque Strategy for Sustainable Environmental Development.* Available online at: http://www.ingurumena.ejgv.euskadi.net [accessed February 28, 2019].

[B39] Guven G. Sulun Y. (2017). Pre-service teachers’ knowledge and awareness about renewable energy. *Renew. Sustain. Energy Rev.* 80 663–668. 10.1016/j.rser.2017.05.286

[B40] Hair J. F. Jr. Hult G. T. M. Ringle C. Sarstedt M. (2016). *A Primer on Partial Least Squares Structural Equation Modeling (PLS-SEM).* Thousand Oaks, CA: Sage.

[B41] Hair J. F. Sarstedt M. Ringle C. M. Mena J. A. (2012). An assessment of the use of partial least squares structural equation modeling in marketing research. *J. Acad. Mark. Sci.* 40 414–433. 10.1007/s11747-011-0261-6

[B42] Hashim H. H. Denan Z. (2015). Importance of preserving the natural environment in the design schools in Malaysia. *Procedia Soc. Behav. Sci.* 170 177–186. 10.1016/j.sbspro.2015.01.027

[B43] Haynes F. (2002). *The Ethical School: Consequences, Consistency and Caring.* Abingdon: Routledge.

[B44] Henseler J. (2017). Bridging design and behavioral research with variance-based structural equation modeling. *J. Advert.* 46 178–192. 10.1080/00913367.2017.1281780

[B45] Henseler J. Hubona G. Ray P. A. (2016). Using PLS path modeling in new technology research: updated guidelines. *Ind. Manage. Data Syst.* 116 2–20. 10.1108/imds-09-2015-0382

[B46] Henseler J. Ringle C. M. Sarstedt M. (2015). A new criterion for assessing discriminant validity in variance-based structural equation modeling. *J. Acad. Mark. Sci.* 43 115–135. 10.1007/s11747-014-0403-8

[B47] Heyl M. Moyano Díaz E. Cifuentes L. (2013). Environmental attitudes and behaviours of college students: a case study conducted at a Chilean university. *Rev. Latinoam. Psicol.* 45 487–500.

[B48] Hitzhusen G. E. (2005). Understanding the role of spirituality and theology in outdoor environmental education: a mixed-method characterization of 12 Christian and Jewish outdoor programs. *Res. Outdoor Educ.* 7 39–56.

[B49] Hitzhusen G. E. (2006). Religion and environmental education: building on common ground. *Can. J. Environ. Educ.* 11 9–25.

[B50] Hofman M. (2015). What is an education for sustainable development supposed to achieve e a question of what, how and why. *J. Educ. Sustain. Dev.* 9 213–228. 10.1177/0973408215588255

[B51] Hossain M. M. Purohit N. (2018). People’s voice to reduce global environmental risks. *Lancet Planet. Health* 2:e333 10.1016/s2542-5196(18)30164-5 30082047

[B52] Hu L. T. Bentler P. M. (1998). Fit indices in covariance structure modeling: sensitivity to underparameterized model misspecification. *Psychol. Methods* 3 424–453. 10.1037/1082-989x.3.4.424

[B53] Hungerford H. R. Volk T. L. (1990). Changing learner behavior through environmental education. *J. Environ. Educ.* 21 8–21. 10.1080/00958964.1990.10753743

[B54] Hussain S. Fangwei Z. Siddiqi A. Ali Z. Shabbir M. (2018). Structural equation model for evaluating factors affecting quality of social infrastructure projects. *Sustainability* 10:1415 10.3390/su10051415

[B55] Ideland M. Malmberg C. (2015). Governing ‘eco-certified children’ through pastoral power: critical perspectives on education for sustainable development. *Environ. Educ. Res.* 21 173–182. 10.1080/13504622.2013.879696

[B56] Jensen B. B. Schnack K. (2006). The action competence approach in environmental education. *Environ. Educ. Res.* 12 471–486. 10.1080/13504620600943053

[B57] Johnson B. (2002). On the spiritual benefits of wilderness. *Int. J. Wilderness* 8 28–32.

[B58] Johnson B. Manoli C. (2011). The ENV scale in the US: a measure of Children’s environmental attitudes based on the theory of ecological attitude. *J. Environ. Educ.* 42 84–97. 10.1080/00958964.2010.503716

[B59] Kals E. Schumacher D. Montada L. (1999). Emotional affinity toward nature as a motivational basis to protect nature. *Environ. Behav.* 31 178–202. 10.1177/00139169921972056

[B60] Kanagy C. L. Nelsen H. M. (1995). Religion and environmental concern: challenging the dominant assumptions. *Rev. Relig. Res.* 37 33–45.

[B61] Kates R. W. Parris T. M. Leiserowitz A. A. (2005). What is sustainable development? Goals, indicators, values, and practice. *Environ. Sci. Policy Sustain. Dev.* 47 8–21. 10.1080/00139157.2005.10524444

[B62] Kellert S. R. Farnham T. J. Farnham T. (2002). *The Good in Nature and Humanity: Connecting Science, Religion, and Spirituality with the Natural World.* Washington, DC: Island Press.

[B63] Kuhlman T. Farrington J. (2010). What is sustainability? *Sustainability* 2 3436–3448. 10.3390/su2113436

[B64] Lambrechts W. Mula I. Ceulemans K. Molderez I. Gaeremynck V. (2013). The integration of competences for sustainable development in higher education: an analysis of bachelor program in management. *J. Clean. Prod.* 48 65–73. 10.1016/j.jclepro.2011.12.034

[B65] Loe Spanish National Education System (2006). *LOE, Spanish National Education System.* Available online at: http://www.boe.es [accessed May 03, 2019].

[B66] LOGSE (1990). *General Organic Law about the Education System in Spain, LOGSE.* Available online at: http://www.boe.es [accessed January 31, 2019].

[B67] Lozano R. (2006). Incorporation and institutionalization of SD into universities: breaking through barriers to change. *J. Clean. Prod.* 14 787–796. 10.1016/j.jclepro.2005.12.010

[B68] Madhawa Nair S. Rashid Mohamed A. Marimuthu N. (2013). Malaysian teacher trainees’ practices on science and the relevance of science education for sustainability. *Int. J. Sustain. High. Educ.* 14 71–89. 10.1108/14676371311288967

[B69] Mat Said A. Ahmadun F. R. Paim L. H. Masud J. (2003). Environmental concerns, knowledge and practices gap among Malaysian teachers. *Int. J. Sustain. High. Educ.* 4 305–313. 10.1108/14676370310497534

[B70] Melkert A. A. Vos R. (2008). *Millenium Development Goal 8: Global Partnership for Achieving the Millenium Development Goals, MDG GAP TASK FORCE REport 2008.* New York, NY: United Nations Secretariat.

[B71] Meyfroidt P. (2013). Environmental cognitions, land change, and social–ecological feedbacks: an overview. *J. Land Use Sci.* 8 341–367. 10.1080/1747423x.2012.667452

[B72] Miles R. Harrison L. Cutter-Mackenzie A. (2006). Teacher education: a diluted environmental education experience. *Aust. J. Environ. Educ.* 22 49–59. 10.1017/s0814062600001658

[B73] Mogensen F. Mayer M. (eds) (2005). “(Eco)-schools: trends and divergences,” in *A Comparative Study on ECO-School Development Processes in 13 Countries* (Vienna: Austrian Federal Ministry of Education, Science and Culture).

[B74] Morrison M. Duncan R. Parton K. (2015). Religion does matter for climate change attitudes and behavior. *PLoS One* 10:e0134868 10.1371/journal.pone.0134868 PMC4527763 26247206

[B75] Murga-Menoyo M. (2009). Educating for local development and global sustainability: an overview in Spain. *Sustainability* 1 479–493. 10.3390/su1030479

[B76] Nekhoroshkov A. V. (2016). Moral dimensions of youth’s world view in multi-ethnic environment. *Int. Electron. J. Math. Educ.* 11 1761–1771.

[B77] Northcott M. (2009). Loving scripture and nature. *J. Study Relig. Nat. Cult.* 3 247–253.

[B78] Okur-Berberoglu E. (2015). The effect of ecopodagogy-based environmental education on environmental attitude of in-service teachers. *Int. Electron. J. Environ. Educ.* 5 86–110. 10.1016/j.evalprogplan.2011.11.007 22321703

[B79] Olsson D. Gericke N. Boeve-de Pauw J. Berglund T. Chang T. (2019). Green schools in Taiwan–Effects on student sustainability consciousness. *Glob. Environ. Chang.* 54 184–194. 10.1016/j.gloenvcha.2018.11.011

[B80] Palmer M. (2008). *Alliance of Religions and Conservation.* Bath: ARC Press.

[B81] Parker L. (2017). Religious environmental education? The new school curriculum in Indonesia. *Environ. Educ. Res.* 23 1249–1272. 10.1080/13504622.2016.1150425

[B82] Parris T. M. (2002). Environmental Education Resources for Grades K–12. *Environment* 44 3–4. 10.1080/00139157.2002.10543557

[B83] Pascual J. A. Esteban G. Martínez R. Molina J. Ramirez J. (2000). La integración de la educación ambiental en la ESO: datos para la reflexión. *Enseñ. Cienc.* 18 227–234.

[B84] Pitoska E. Lazarides T. (2013). Environmental education centers and local communities: a case study. *Procedia Technol.* 8 215–221. 10.1016/j.protcy.2013.11.030

[B85] Ponomarenko Y. V. Zholdasbekova B. A. Balabekov A. T. Kenzhebekova R. I. Yessaliyev A. A. Larchenkova L. A. (2016). Modern methodology and techniques aimed at developing the environmentally responsible personality. *Int. J. Environ. Sci. Educ.* 11 2877–2885.

[B86] Pope Paul, VI (1971). *Apostolic Letter Octogesima Adveniens.* Rome: AAS.

[B87] Ramos T. B. Montano M. Joanaz, de Melo J. Souza M. P. Carvalho (2015). Strategic environmental assessment in higher education: portuguese and brazilian cases. *J. Clean. Prod.* 106 222–228. 10.1016/j.jclepro.2014.12.088

[B88] Rauch F. (2002). The potential of education for sustainable development for reform in schools. *Environ. Educ. Res.* 8 43–51. 10.1080/13504620120109646

[B89] Raven P. H. I. (2016). Our World and Pope Francis’. Encyclical, Laudato si’. *Q. Rev. Biol.* 91 247–260. 10.1086/688094 29558610

[B90] Rickenbacker H. Brown F. Bilec M. (2019). Creating environmental consciousness in underserved communities: Implementation and outcomes of community-based environmental justice and air pollution research. *Sustain. Cities Soc.* 47:101473 10.1016/j.scs.2019.101473

[B91] Rigdon E. E. Sarstedt M. Ringle C. M. (2017). On comparing results from CB-SEM and PLS-SEM: five perspectives and five recommendations. *Mark. Zfp* 39 4–16. 10.15358/0344-1369-2017-3-4

[B92] Ringle C. M. Wende S. Becker J. M. (2015). *SmartPLS 3.* Boenningstedt: SmartPLS GmbH.

[B93] Rittel H. W. Webber M. M. (1973). Dilemmas in a general theory of planning. *Policy Sci.* 4 155–169. 10.1007/bf01405730

[B94] Robina Ramírez R. Fernández Portillo A. (2018). What role does touristś educational motivation play in promoting religious tourism among travellers? *Ann. Leis. Res.* 1–22. 10.1080/11745398.2018.1561309

[B95] Robina-Ramírez R. Medina-Merodio J.-A. (2019). Transforming students’ environmental attitudes in schools through external communities. *J. Clean. Prod.* 232 629–638. 10.1016/j.jclepro.2019.05.391

[B96] Robina-Ramírez R. Merodio J. A. M. McCallum S. (2020). What role do emotions play in transforming students’ environmental behaviour at school? *J. Clean. Prod.* 258:120638 10.1016/j.jclepro.2020.120638

[B97] Robina-Ramírez R. Pulido Fernández M. (2018). Religious Travellers’ Improved Attitude towards. *Nat. Sustain.* 10:3064 10.3390/su10093064

[B98] Robina-Ramírez R. Pulido-Fernández M. (2019). What role do religious belief and moral emotions play in pilgrimage with regards to respecting nature? *Ann. Leis. Res.* 1–21. 10.1080/11745398.2019.1703199

[B99] Sánchez-Llorens S. Agulló-Torres A. Del Campo-Gomis F. J. Martinez-Poveda A. (2019). Environmental consciousness differences between primary and secondary school students. *J. Clean. Prod.* 227 712–723. 10.1016/j.jclepro.2019.04.251

[B100] Sarstedt M. Hair J. F. Ringle C. M. Thiele K. O. Gudergan S. P. (2016). Estimation issues with PLS and CBSEM: Where the bias lies! *J. Bus. Res.* 69 3998–4010. 10.1016/j.jbusres.2016.06.007

[B101] Schreiner P. (ed.) (2000). *Religious Education in Europe. A Collection of Basic Information about RE in European Countries.* Münster: Intereuropean Commission on Church and School and Comenius-Institut.

[B102] Shindler B. A. Cramer L. A. (1999). Shifting public values for forest management: making sense of wicked problems. *West. J. Appl. For.* 14 28–34. 10.1093/wjaf/14.1.28

[B103] Shwom R. Lorenzen J. A. (2012). Changing household consumption to address climate change: social scientific insights and challenges. *Wiley Interdiscip. Rev. Clim. Chang.* 3 379–395. 10.1002/wcc.182

[B104] Singseewo A. (2011). Awareness of environmental conservation and critical thinking of the undergraduate students. *Eur. J. Soc. Sci.* 25 136–144.

[B105] Sponsel L. E. (2012). *Spiritual Ecology: A Quiet Revolution.* Santa Barbara, CA: ABC-CLIO.

[B106] Stone M. (1974). Cross-validatory choice and assessment of statistical predictions. *J. R. Stat. Soc. Ser. B* 36 111–133. 10.1111/j.2517-6161.1974.tb00994.x

[B107] Svanström M. Lozano-Garzia F. J. Rowe D. (2008). Learning outcomes for sustain-able development in higher education. *Int. J. Sustain. High. Educ.* 9 339–351.

[B108] Tal T. (2010). Pre-service teachers’ reflections on awareness and knowledge following active learning in environmental education. *Int. Res. Geogr. Environ. Educ.* 19 263–276. 10.1080/10382046.2010

[B109] Torkar G. Krašovec U. (2019). Students’ attitudes toward forest ecosystem services, knowledge about ecology, and direct experience with forests. *Ecosyst. Serv.* 37:100916 10.1016/j.ecoser.2019.100916

[B110] Tucker M. E. (2009). “Touching the depths of things: cultivating nature in East Asia,” in *Ecology and the Environment: Perspectives from the Humanities*, ed. Swearer D. (Cambridge, MA: Harvard Center for the Study of World Religions), 49–64.

[B111] Tucker P. (1999). Normative influences in household waste recycling. *J. Environ. Plan. Manag.* 42 63–82. 10.1080/09640569911307

[B112] UN (2009). *Agenda 21.* Available online at: http://www.un.org/esa/dsd/agenda21/ [accessed December 07, 2019].

[B113] UNECE (2005). *UNECE Strategy for Education for Sustainable Development CEP/AC.13/2005/3/Rev1.* Paris: UN Economic and Social Council.

[B114] UNESCO (1977). *The Tbilisi Declaration – Intergovernmental Conference on Environmental Education Final Report.* Tbilisi: USSR.

[B115] Uzzell D. L. Rutland A. Whistance D. (1995). “Questioning Values in Environmental Education,” in *Values and the Environment*, eds Guerrier Y. Alexander N. Chase J. O’Brien M. (Chichester: Wiley), 172–182.

[B116] Valderrama-Hernández R. Alcántara L. Limón D. (2017). The complexity of environmental education: teaching ideas and strategies from teachers. *Procedia Soc. Behav. Sci.* 237 968–974. 10.1016/j.sbspro.2017.02.137

[B117] Van Eijndhoven J. Clark W. C. Jäger J. (2001). “The long-term development of global environmental risk management: conclusions and implications for the future,” in *Learning to Manage Global Environmental Risks: A Comparative History of Social Responses to Climate Change, Ozone Depletion, and Acid Rain*, Vol. 1 eds Clark W. C. Jaeger J. van Eijndhoven J. Dickson N. M. (Cambridge, MA: The MIT Press), 181–197.

[B118] Van Petegem P. Blieck A. Pauw J. B. D. (2007). Evaluating the implementation process of environmental education in pre-service teacher education: two case studies. *J. Environ. Educ.* 38 47–54. 10.3200/JOEE.38.1.47-54519146

[B119] Vare P. Scott W. (2007). Learning for a change: exploring the relationship between education and sustainable development. *J. Educ. Sustain. Dev.* 1 191–198. 10.1177/097340820700100209

[B120] Vázquez C. E. Sevillano García M. L. (2011). *Programar en Primaria y Secundaria.* Madrid: Pearson.

[B121] Ward M. N. Wells B. Diyamandoglu V. (2014). Development of a framework to implement a recycling program in an elementary school. *Resour. Conserv. Recycl.* 86 138–146. 10.1016/j.resconrec.2014.02.013

[B122] WCED (1987). *Our Common Future.* London: Oxford University Press.

[B123] World Health Organization [WHO] (2013). *Urban Population Growth.* Available online at: http://www.who.int [accessed February 6, 2019].

[B124] Yildirim A. Baştuğ İ (2010). Teachers’ views about ethical leadership behaviors of primary school directors. *Procedia Soc. Behav. Sci.* 2 4109–4114. 10.1016/j.sbspro.2010.03.648

[B125] Ysseldyk R. Matheson K. Anisman H. (2010). Religiosity as identity: toward an understanding of religion from a social identity perspective. *Pers. Soc. Psychol. Rev.* 14 60–71. 10.1177/1088868309349693 20089847

[B126] Zembylas M. (2007). Emotional ecology: the intersection of emotional knowledge and pedagogical content knowledge in teaching. *Teach. Teach. Educ.* 23 355–367. 10.1016/j.tate.2006.12.002

